# Hot executive control and response to a stimulant in a double-blind randomized trial in children with ADHD

**DOI:** 10.1007/s00406-016-0683-8

**Published:** 2016-03-10

**Authors:** Jessica Yarmolovsky, Tamar Szwarc, Miguel Schwartz, Emanuel Tirosh, Ronny Geva

**Affiliations:** 1 The Gonda Brain Research Center, Bar Ilan University, Ramat Gan, Israel; 2 Department of Psychology, Bar Ilan University, Ramat Gan, Israel; 3 The Hannah Khoushy Child Development Center, TheBnei Zion Medical Center, Haifa, Israel; 4 The Bruce Rappaport Faculty of Medicine, The Technion, Israel Institute of Technology, Haifa, Israel

**Keywords:** Methylphenidate, ADHD, Executive control, Children

## Abstract

Attention deficit hyperactivity disorder (ADHD) is thought to involve an executive inhibitory control (IC) deficit, yet it is not clear if this is a general deficit affecting both cold and hot EC, and if methylphenidate (MPH) affects both systems in treated children. We explored this by using a Stroop-like task in children with ADHD as compared to controls, containing different types of emotional stimuli (six levels), and we investigated the role of intervention with MPH on IC as compared to placebo. Children with ADHD and controls (*N* = 40; 7–13 years old) were tested with a hot and cold Stroop-like task. This was followed by a double-blind placebo-controlled crossover trial of the effect of MPH on these skills. Children with ADHD showed a specific difficulty inhibiting their responses, particularly to hot, angry and frustration-inducing stimuli. Further, treatment with MPH was effective in reducing errors toward frustration-inducing stimuli as compared to placebo (*p* < .05, *η* ^2^ = .347), particularly with repeated exposure to the stimuli. Results indicate that children with ADHD experience executive control difficulties, particularly in hot negative emotional contexts. These emotion regulation difficulties are amenable to stimulant intervention.

## Introduction

Theories about attention deficit hyperactivity disorder (ADHD) emphasize a neonatal origin [1] that compromises information processes [2], with an evolving role for executive dysfunctions in children [3, 4]. Specifically, neurocognitive models of ADHD posit that executive control (EC) is a core deficit [5–7], while others point to deficits in emotional control and motivational processes [8, 9]. The relationship between executive attention and emotional control in ADHD may have pervasive effects on patients’ well-being in manners that have not yet been fully elucidated.

Difficulties processing information have been suggested as an explanation for the impulsivity that is characteristic of ADHD [10]. Research has shown that information processing, regardless of load type, is impaired in children with ADHD [7], providing possible insight into the origin of executive dysfunction in this population. One such executive deficit is inhibitory control (IC), a person’s ability to inhibit a dominant response in order to activate a different, more appropriate one [11]. IC is thought to play an important role in self-regulation [6], adapting to environmental changes, learning, planning and decision-making [12–14]. Recent studies exploring IC impairments in ADHD yielded varied results, with some reporting no difference between ADHD and controls [15], and others highlighting ADHD IC deficits [16]. It is plausible that the discrepancies noted may be explained in part by differences in emotional valence embedded in various inhibitory tasks, and by differences in task demands in their degree of activation of the emotional regulation system. That is, some tasks activate emotional responses, such as a happy, sad, or anger–frustration more so than others, as a function of their content and the level of success or failure experienced while performing each task.

Indeed, neuropsychological literature and recent neuroscience data point to at least two distinct executive networks that are active already in early development [17, 18]; one that is sensitive to conceptual rules and symbolic target-oriented behavior, the “cold executive network”; and the other, a motivational system that is more dependent on social-affective information and reward, the “hot executive network” [19]. These two networks have been thought to involve some shared and distinct neural structures [20] and to have differential developmental pathways [21–23].

Neural network differences in this regard are such that the cold system, responsible for updating, inhibition and flexibility, seems to activate the dorsolateral prefrontal cortex (DLPFC), along with mid-superior frontal gyrus, superior and inferior temporal gyrus and inferior parietal cortex network. The hot system on the other hand, enabling self-regulation, decision-making and emotion perception, activates more ventromedial and orbital prefrontal cortex areas [24, 25], along with some shared activity in the DLPFC, mid-superior frontal gyrus, anterior and mid-posterior cingulate, and temporal and fusiform gyrus [20].

Research concentrating on the interrelations between these networks suggests that hot and cool EF rely on some shared and some different, yet interconnected prefrontal circuitry, with hot EF tasks involving moderation and interference from affective and reward processing regions, mostly through thalamic-limbic ventral striatum circuitry [19, 26]. The literature has largely concentrated on different tasks to probe each network discretely [26]. It may be useful to probe both components using the same task, to better target the specific EF deficit experienced by children with ADHD.

Imaging data in children with ADHD suggest the involvement of both DLPFC and ventromedial prefrontal cortex (vmPFC) networks, along with these networks’ subcortical structures [27]. It has also been suggested that the vmPFC is associated with impulse control, risk-taking [28, 29], emotional regulation and emotional reward-driven motivation [30, 31], pointing to the potential for a marked involvement of this network in ADHD. Indeed, adolescents with ADHD showed increased medial prefrontal cortex reactivity during an emotional processing task as compared to controls, while no differences were noted between ADHD and controls in response to a cold task [32]. This possibly points to an ADHD-related difference in the hot system as compared to the cold one.

The idea that the hot system is affected in ADHD is further supported by reports that explore this relationship on a behavioral level. Children and adolescents with ADHD experience deficits in processing emotional content [33–38], particularly stimuli with angry valence [39, 40]. Further, childhood hot EF is considered a fairly stable trait [41] that has been related to long-term deficits in interpersonal relations [42] and stress mismanagement [43].

Integration of the above literature seems to point to the notion that executive control differences in ADHD may be particularly susceptible to tasks with hot content, possibly more so with negative valence, at school-age years. To examine this notion, and yet limit task impurity concerns [44], the current study incorporated emotional context in one inhibitory task in preadolescent participants with ADHD [21]. Expectations were such that school-aged children with ADHD would experience more difficulties in response to hot valenced stimuli as compared to an equivalent cold EC using a simple Stroop-like task with non-emotionally loaded stimuli, as well as stimuli with added neutral and emotional facial expressions [45]. To examine this notion further, we then explored the efficacy of MPH as a function of emotional valence in children with ADHD using a placebo-controlled crossover design.

### Effect of methylphenidate (MPH) on EC to emotionally loaded stimuli

MPH, the most common treatment for ADHD, operates by blocking dopamine-active reuptake transporter and increasing extracellular dopamine proportionally to the level of blockade and to the rate of dopamine release [46]. This process is associated with a boost in the noradrenergic system, evident by increased activity in the frontal lobe, an enhanced perception of the external stimuli in patients with ADHD [47], and improved arousal alertness and normalization of otherwise immature intracortical inhibition [48, 49].

MPH has been shown to improve fine motor coordination, reaction time, memory, response inhibition, vigilance, impulse control, attention, concentration [50], as well as to support social cognitive and academic functioning in children with ADHD [51–53], though its efficacy on executive control in healthy cohorts yields small yet significant results [54], while effects in children with ADHD are inconclusive [55] and non-uniform [56, 57]. These findings may be partially due to differential activation of hot and cold networks in different executive attention tasks. That is, MPH was found to be effective in treating emotional processing and reward-driven deficits in children with ADHD [58–60], suggesting that stimulant treatment would be more effective in IC tasks that elicit frustration or emotional responses.

Comparisons of cold and hot executive effects in ADHD are often limited by task impurity (i.e., the use of different tasks to evaluate different EC constructs [44]), making it particularly difficult to compare cold and hot functions [8, 9, 21, 22]. Thus, it has been difficult to distinguish whether noted differences stem from EC type, or rather differences are due to task variability. The current study therefore examined cold and hot EC in 7- to 13-year-old children with ADHD with and without pharmacological intervention (none, placebo, MPH), as compared to controls, using the same simple Stroop-like inhibitory task [61] with and without added emotional conditions [45].

The paradigm enabled the investigation of two hypotheses: First, in 7- to 13-year-old children, the hot EC task, especially angry or frustration-inducing emotions, would be demanding for children with ADHD, as compared to the cold task.

Second, MPH, as compared to no intervention and placebo, would moderate IC deficits more effectively, particularly in regulating responses that probe the hot network, by affecting arousal regulation, with an emphasis on negative valence.

## Methods

The current research explored ADHD effects on cold and hot EC in children with ADHD as compared to age-matched controls, and then employed a double-blind placebo-controlled crossover study investigating the effect of MPH on children with ADHD who underwent the same task.

### Participants

Forty 7- to 13-year-old participants (mean age = 11.2 ± 1.4 years; 42 % female) were enrolled in the study and classified into ADHD (*N* = 20) and non-ADHD (*N* = 20) groups. All ADHD participants were referred by their community pediatricians for evaluation as required by the ministry of health guidelines. ADHD diagnosis was made by a pediatric neurologist specializing in ADHD in the child psychiatric department unit at the hospital according to the protocol employed in the clinic. Protocol included clinical evaluations, the Child Behavior Check List [62] and the DSM-IV-TR symptom questionnaire [63]. Control participants were randomly recruited from mainstream public schools in the same (central) district of Israel via word-of-mouth (snowball recruitment) and were exposed to the same educational curriculum. Their past medical history, as reported by their parents, was typical. All participants came from middle socioeconomic two-parent families. Exclusion criteria included: history of chronic mental illness other than attention or organization dysfunctions, diagnosed neurological disorders, learning disorders, social/emotional difficulties not characteristic of ADHD according to the DSM-IV, and intelligence within one standard deviation from the mean for chronological age. Given its known comorbidity in ADHD, conduct disorder was not selected as an excluding factor among the ADHD group; however, in the current sample, only one case scored in the clinical range of aggression on the CBCL questionnaire. This child’s data were explored due to a potential outlier concern, but he did not show a unique profile on the task. No gender differences were seen across groups, and distribution replicated ADHD in the general population (female gender ADHD = 35 %, non-ADHD = 65 %, *p* = .200). Age differences between groups were nearly significant (ADHD = 11.25 ± 1.5, non-ADHD = 11.6 ± 1.2, *p* = .077); thus, participant age was held as a covariate throughout analysis. All children included in the placebo-controlled crossover design were candidates for intervention with MPH and were medication naive.

### Procedure

The study was approved by the Helsinki review boards at Bnei Zion Hospital and at Bar Ilan University. All parents of participants signed written informed consent, and children expressed oral consent prior to participation.

### Intervention procedure

The ADHD group was presented with the experimental task three times, first without medication, followed by two randomly ordered intervention conditions: (1) MPH (10 mg, Ritalin); and (2) placebo look a-like capsules, prepared by the pharmacy at Bnei Zion hospital. MPH or placebo pills were administered at the Pediatric Neurology clinic by a medical team member who was blind to the pill content. Time lags between sessions were about a week (between sessions one and two, mean 9.6, SE 2.2 days; and between sessions two and three, mean 6.6, SE 1.1, paired samples *T* = 1.362, *p* = .190, NS).

### The emotional day night task (EDN)

The emotional day night (EDN) [45] is a computerized Stroop-like task adapted from the day–night inhibitory response task (DN) [64], modified to incorporate socioemotional blocks. Strong test–retest reliability was previously reported for the day–night task in a study conducted on 7- to 12-year-old participants [65], and the EDN adaptation was reported to be effective in probing IC in typical young adults [45].

A survey was conducted to ensure validity of emotional stimuli using an open-answer format questionnaire in which typically developing participants (*N* = 34) noted the emotion expressed in each stimulus. Results yielded high agreement for all stimuli.^1^

**Fig. 1:**
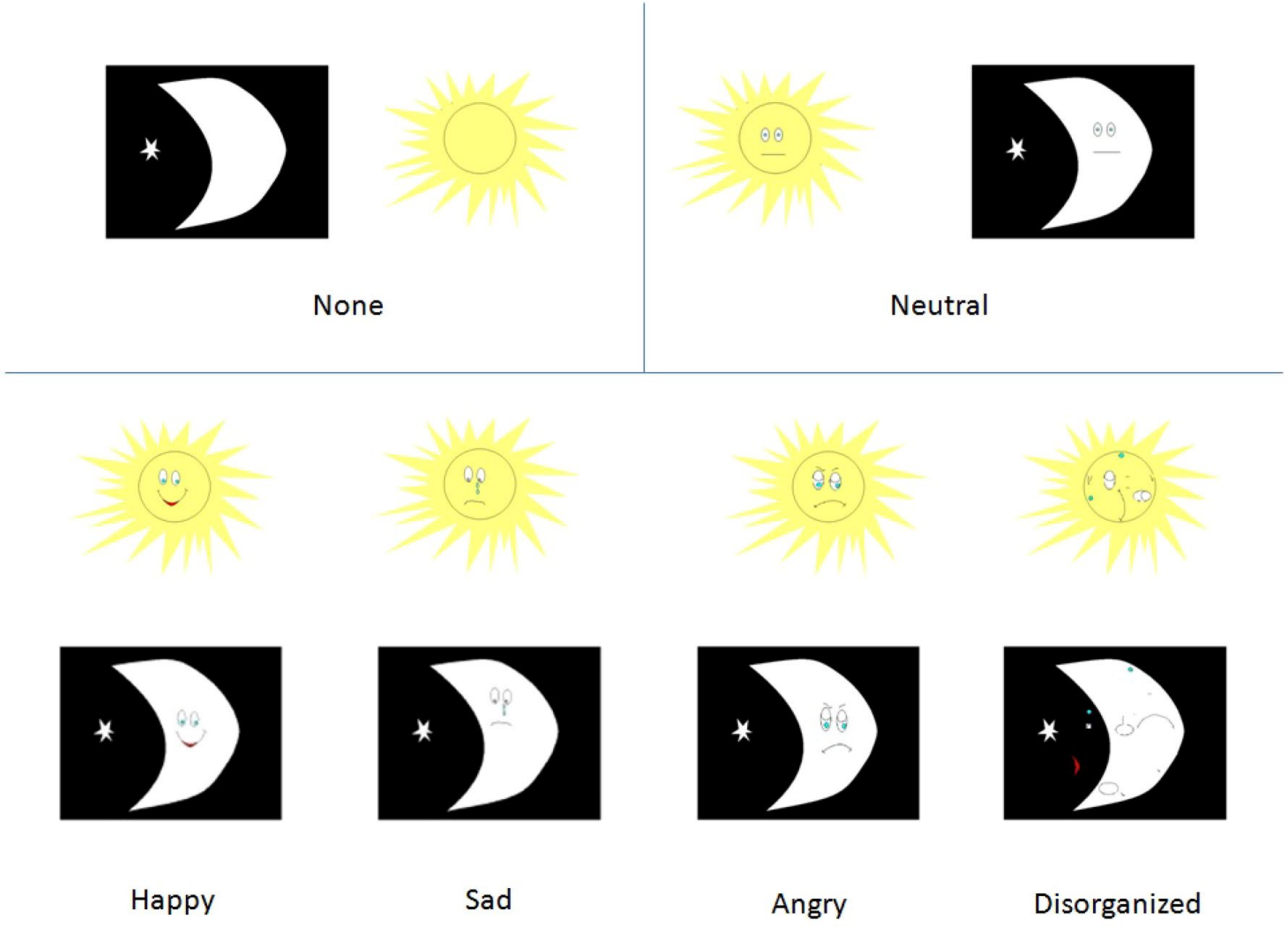
EDN stimuli set

The first block of the EDN consists of 16 practice trials in which participants are asked to click a “sun” or a “moon” mouse key corresponding to an image that appears on screen. The second block activates an inhibitory control component, during which participants are instructed to choose the opposite image to what appears on screen, and in addition to the no-load sun and moon images, added socioemotional stimuli are incorporated within the stimuli. This block consists of 112 randomized trials of no-load, neutral, happy, sad, angry and disorganized stimuli (Fig. 1). Each emotion type is presented 16 times (8 moons and 8 suns). The trial procedure consists of a fixation cross presented for 800 ms, a 100-ms pause, and 120 ms of stimulus presentation, followed by an additional 1000 ms for response.

### Data analysis

Descriptive statistics were analyzed to ensure task difficulty. Next, a repeated-measures analysis was conducted to compare error rate differences in ADHD and control groups toward no-load, neutral, happy, sad, angry and disorganized stimuli. Finally, to explore intervention effects, differences between no intervention and intervention conditions were calculated and a repeated-measures analysis was run comparing error rate differences in the two intervention conditions (MPH, placebo) and presentation order was held as a between-subjects variable. Age and aggressive behavior were held as covariates throughout analysis due to near-significant group differences for age and propensity for increased conduct disorder in children with ADHD [66]. Analysis was conducted using IBM SPSS statistics package 20.

## Results

Results were first analyzed to ensure task difficulty was appropriate for all participants. Descriptive statistics showed that overall all children did err to some degree on this task. Average percent errors for the participants were 23 ± 14, suggesting the task was effective for the population and for the research aims.

### ADHD effect on hot and cold EC

**Fig. 2:**
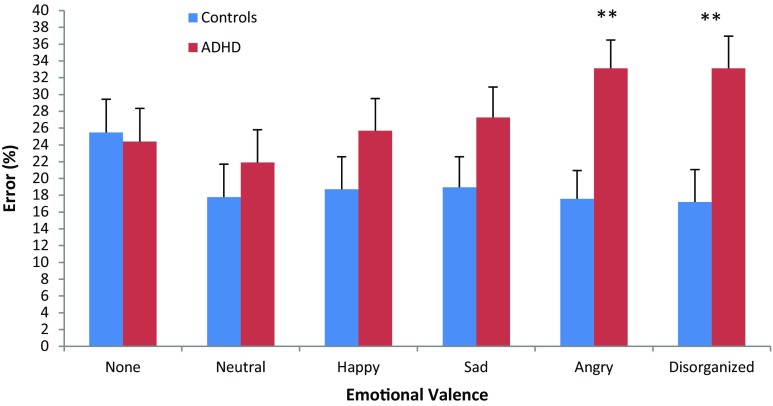
Error rates (%) as a function of emotional valence and group. *Legend* **p < .01

To explore ADHD differences in response to each stimulus type, repeated-measures analysis was conducted comparing cold, neutral, happy, sad, angry and disorganized stimuli as a function of ADHD, with age and aggression held as covariates. Results yielded an emotional valence by ADHD group interaction effect [*F*_(1, 36)_ = 3.292, *p* < .05, *η* ^2^ = .340] (Fig. 2), such that as the content became emotional, particularly negatively loaded, group differences increased. Post hoc analysis shows specific effects for stimuli with angry and disorganized valence [angry *F*_(1,36)_ = 10.884, *p* < .002, *η* ^2^ = .232; disorganized *F*_(1,36)_ = 9.330, *p* < .004, *η* ^2^ = .206].

### Intervention × stimulus effect on EC

To assess overall intervention effects, repeated-measures analyses were run comparing total error rate as a function of intervention type. Results showed increased errors in the non-intervention condition, as compared to both MPH [*F*_(1,19)_ = 12.621, *p* < .01, *η* ^2^ = .399] and placebo [*F*_(1,19)_ = 12.758, *p* < .01, *η* ^2^ = .415] conditions. In order to account for potential learning affects, we computed differences between errors made in the non-medication condition administered first, and the intervention conditions.

**Fig. 3:**
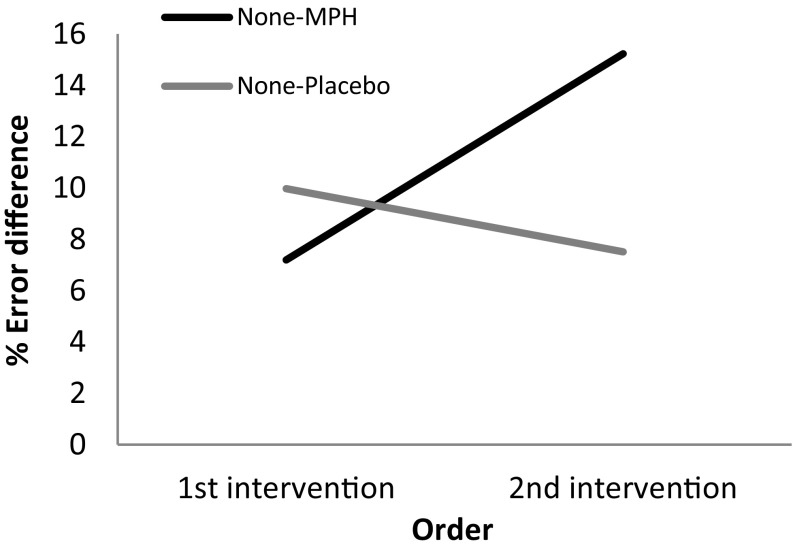
Error differences (%) as a function of intervention type and intervention order

In assessing differences between MPH and placebo interventions, a repeated-measures analysis was conducted with valence (none, neutral, happy, sad, angry and disorganized) and intervention difference score (MPH-none, placebo-none) as within-subject variables, and presentation order as a between-subjects variable. Age and aggression scores were held as covariates. Results indicated an intervention by order effect [*F*_(1,19)_ = 5.474, *p* < .05, *η* ^2^ = .267] (Fig. 3), and no main effects. The interaction was such that increased differences in error rates were noted among MPH intervention conditions as exposure to stimuli increased, while an opposite trend occurred in response to placebo.

**Fig. 4:**
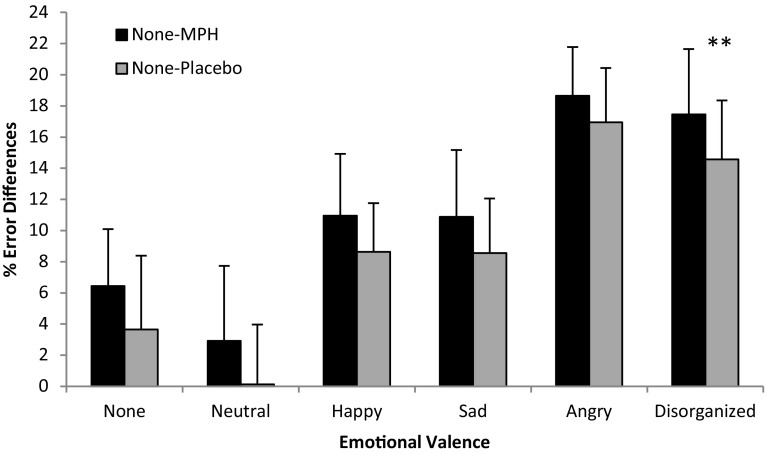
Error differences (%) as a function of intervention type and emotional valence. *Legend* **p < .01

Additionally, a moderate-strong emotional valence × intervention interaction effect was noted [*F*_(1,15)_ = 3.521, *p* < .05, *η* ^2^ = .615] (Fig. 4), with a general trend of increased percent error reductions in MPH as compared to placebo. Post hoc analysis indicates that this intervention effect amounts to a significant level specifically in response to disorganized stimuli [*F*_(1,15)_ = 9.708, *p* < .01, *η* ^2^ = .393].

Finally, the same model as above was applied to evaluate response times (RT) to correct answers in the task. The model included age and aggression as covariates. Results yielded group differences in responding to the happy condition [mean ± SD, for ADHD 605.88 ± 197.68, controls 679.87 ± 228.73; *F*_(1,37)_ = 4.384, *p* < .05, *η* ^2^ = .114], with children in the ADHD group responding more quickly than in the controls in this condition. Further intervention effect analyses, controlling for intervention order, along with the above covariates, yielded null results.

## Discussion

This study examined the notion that children with ADHD have more difficulty exerting inhibition in hot contexts, specifically those that induce anger and frustration. We explored this by investigating EF in a non-emotional and emotionally loaded inhibition task in 7- to 13-year-old children with ADHD as compared to controls, and further validated the notion by exploring MPH intervention effects using a double-blind placebo-controlled crossover design.

In this fairly simple Stroop-like task, no ADHD main effect was noted in response to the cold stimuli. Results in the current report, with a simple low-frustration go task, show that cold IC in children with ADHD is preserved, yet when confronted with specific emotional valence, performance is compromised. Indeed, previous studies with 6- to 13-year-old children found no differences between ADHD and controls in response to a Stroop task [15] that included non-emotional stimuli, as was seen in the current paradigm in response to the cold stimuli.

By using one task to explore both cold and emotional stimuli, differences emerged between ADHD and controls in their error rates to angry and disorganized expressions, and in their response rates to the happy stimuli. Two potential interpretations may be considered with regard to this finding. The first may point to a particular deficit in the hot as compared to the cold EF system, activated by highly arousing negative stimuli. A specific deficit in hot EF has been noted in children with susceptible attention networks [17, 67]. Integrating current findings with the latter literature may suggest that the hot EF system is more susceptible in populations whose attention skills and or their self-regulation abilities are deficient.

A somewhat different account may rely on the interdependence between the two EF networks. Cunningham and Zelazo’s neurological iterative reprocessing model [68, 69] suggests that hot and cold EF encompass a continuum in which relatively hot and cool aspects interact with one another sequentially. This enables initial thalamus–amygdala-driven basic hot approach–avoidance motivational trends, which are then reprocessed, prefrontally by the OFC and the vmPFC, enabling reflection and behavior regulation according to a more cool complex hierarchy of rules, with the latter shown to be deficient in ADHD [27].

Research has suggested that high arousing negative stimuli are particularly potent in activating initial avoidance, while sad and happy stimuli promote a less clear approach/avoidance pattern [70]. Taken together, current results suggest that children with ADHD show difficulty regulating EF in anger and frustration contexts. These data extend the current focus on inhibitory control deficits in ADHD by underscoring an ADHD compromise in exerting EF in contexts that require management of initial avoidance motivation in “go” tasks.

The current difficulties in angry and frustration-inducing stimuli may contribute to the framework that suggests a unitary EF [71], with negative and ambiguous affective content possibly signaling a greater challenge in suppressing “go” drives. This account seems to fit well with the data, particularly since the task did not involve a demand for delay of gratification, but rather a “go” task as theoretically postulated [71, 72].

Findings also resonate with reports from ecological settings of children with ADHD. Research notes deficits in emotional processing in children with ADHD [32] with regard to negative angry stimulation [37]. This pattern has been credited to preattentive processing [73] and/or an attentional bias during IC tasks. According to this notion, emotional processing acts as a distraction and consumes mental resources [32, 74]. Such an overload may potentially result in a blunted intracellular dopamine response that prevents an organized appropriate response that is needed for the IC challenge [75, 76].

Current findings may also pertain to emotional regulation issues in ADHD. Previous work found that children with ADHD have greater difficulty identifying emotional stimuli, especially negative ones [37, 39, 77]. The current findings may shed light onto the notion that inhibitory control in children with ADHD is particularly susceptible in negatively loaded circumstances. Indeed, frustration-inducing paradigms were shown to elicit behaviors associated with emotional dysregulation in children with ADHD [78].

This deficit may be relevant in understanding social relationships of children with ADHD. Their relationships are often characterized by impulsive responses, increased aggression and conflicts, as compared to children without ADHD [5, 36, 79–81]. This aggressive characteristic has been associated with emotional regulation deficits [78]. Current results, controlling for aggression, suggest that the IC deficits in response to negative emotional contexts are deficient in ADHD irrespective of the ability to inhibit aggressive tendencies. The IC deficit in negative emotional contexts may play a role in social encounters of children with ADHD with their peers, as well as with adults [82], thus perpetuating a cycle of poorly regulated social interactions [33, 82, 83].

The difficulty in executive control toward emotional stimuli seems compatible with imaging reports that suggest disturbances in ventromedial/orbital fronto-amygdala connections in ADHD [84]. Specifically, when participants with ADHD were presented with facial expressions of anger, reduced electrocortical early parieto–occipital activity was documented in children [73] and late right parietal positivity was documented in male adults [85]. These pathways, which are thought to play a key role in orienting and processing emotional stimuli, possibly set a deficient starting point for later phases of executive control while regulating emotional arousal [86] in children with ADHD, deficits which were behaviorally probed in the current study.

These results were also complimented in the current study with pharmacological intervention data. Current data point to an MPH effect when comparing error rates in response to stimuli loaded with different emotions. Responses indicated that children with ADHD benefit from MPH intervention, especially in regulating responses to disorganized stimuli (which were most affected as compared to controls), more so than placebo intervention. This finding further supports the notion that noradrenergic pathways in children with ADHD provide a more optimal base for regulating arousal and recruiting mental resources needed for executive control, particularly in negatively charged [87] or ambiguous conditions [88].

The literature so far has concentrated on repeated exposure to MPH and has understudied the interaction of intervention with repeated exposure of the stimuli, leading to greater levels of familiarity in order to reduce ambiguity aversion [89]. Current results point to MPH’s increased efficacy in such conditions of repetition, which may be relevant in learning environments. These results seem to resonate with the literature in showing that repeated stimulus exposure with MPH may be a promising venue to pursue in providing support for emotional EC difficulties in children with ADHD [78]. Previous literature regarding children with ADHD, particularly in the contexts of learning academic tasks, has documented intervention by order effects in ADHD [90, 91], while others have not [87]. This discrepancy may point to a contextual factor, possibly an emotional one. That is, the effect may be particularly useful in ADHD when stimulus familiarity interacts with negative emotions, which is at the forefront of the executive regulatory challenge for children with ADHD.

Indeed, it has been suggested that efficacy of self-regulation in social interactions of children and young adolescents with ADHD is highly dependent upon contextual factors, and on the micro-sequence of events that proceed the response, particularly when events are negatively loaded [82]. Current data show that stimuli familiarity, particularly negative disorganized ones, is most responsive to stimulant medication, through supporting self-regulation and executive control in children with ADHD [91].

Future work may explore further the influences of different dosages and different exposure durations, and their interaction with negative emotional valence and level of familiarity. This can further deepen our understanding of intervention type by valence interaction of executive regulation in children with ADHD.

## Footnotes

1 Validity survey results note high agreement rates per stimuli as follows: Happy, 100 %; Sad, 97 %; Angry, 94 %; Disorganized, 93 % reported unclear, confused or negative, or eliciting frustration in unknowing what the stimuli represent; Neutral 62 % reported neutral, 20 % rated unspecified emotion and the remaining were equally distributed between negative (12 %) and positive emotions (8 %).
